# Predictors of mortality in a Chinese medical ICU: a focus on ICU-acquired bloodstream infections

**DOI:** 10.1186/s12879-026-12695-y

**Published:** 2026-01-27

**Authors:** Guo Long, Chao Jia, Chunjiao Long

**Affiliations:** 1 Department of Respiratory and Critical Care Medicine, The Third Xiangya Hospital of Central South University, Changsha, 410013 China; 2 Nursing Department, The Third Xiangya Hospital, Central South University, Changsha, Hunan, China; 3 Department of Intensive Care Unit, Qingdao Municipal Hospital, Qingdao University, Qingdao, 266000 China; 4 Department of Nephrology, The Third Xiangya Hospital of Central South University, Changsha, 410013 China

**Keywords:** Risk factors, Mortality, Bloodstream infection, Medical intensive care unit

## Abstract

**Background:**

Bloodstream infection (BSI) is a leading cause of morbidity and mortality among critically ill patients. This retrospective study aimed to determine the frequency of a medical intensive care unit (ICU)-acquired BSIs and identify the mortality risk factors associated with BSIs in critically ill patients within the medical ICU who were transferred from the internal medicine department, not from the surgical department after a surgery.

**Methods:**

Blood samples were processed using the BACTEC 9240 blood culture system, with species identification conducted for culture-positive cases using a Bruker mass spectrometer, and susceptibility tests by Vitek-2 system, or TDR YEAST-AST system. Pre-specified risk factors for mortality were analyzed through logistic regression.

**Results:**

Over a 9-year period, 113 isolates of BSI occurred in 106 (2.2%) of 4820 patients. Gram-negative bacteria predominated as the primary pathogens, accounting for 64.6% of all isolated pathogens. The most frequent pathogen was Enterobacteriaceae (36.3%), primarily *Klebsiella pneumoniae* (25.7%). The lungs were the most common source of infections. Septic shock was present at the onset of BSI in 71 (67.0%) patients. Of the total, 66 (62.3%) patients died within 1 month following the onset of BSIs. Multivariate analysis identified the following independent factors for 1-month all-cause mortality: lymphocyte count < 0.5 × 10⁹/L (OR = 4.305) and septic shock (OR = 3.275), while BMI ≥ 23 (OR = 0.181) and appropriate antibiotic treatment (OR = 0.333) remained protective.

**Conclusions:**

This study, focusing on a high-risk medical ICU population with BSIs, identifies key factors associated with mortality: septic shock as the predominant risk factor, the prognostic relevance of severe lymphopenia, the critical role of appropriate antibiotics, and the potential consideration of the “obesity paradox” (BMI ≥ 23) in patient assessment.

## Background

In recent decades, bloodstream infections (BSIs) have emerged as one of the leading causes of morbidity and mortality among critically ill patients, particularly in older and immunocompromised individuals. These infections predispose patients to multiple organ failure, septic shock, and disseminated intravascular coagulation, and are associated with prolonged stays in the intensive care unit (ICU) as well as significant socioeconomic costs [1–4]. Globally, the annual incidence of BSI is reported at 204 per 100,000 population, with a mortality rate ranging from 15% to 60%, accounting for approximately 10% of ICU inpatients [5–10].

The determinants of mortality in patients with BSIs in general ICUs have been extensively studied. Varon et al. recently conducted a systematic review encompassing 62 studies with over 300,000 patients [11]. They aimed to summarize the factors that were repeatedly demonstrated as associated with mortality in studies evaluating patients with BSIs caused by bacteria, through a systematic review of observational studies to reduce bias, lower heterogeneity and enhance the comparability of studies, so their conclusions reflect global risk factors for mortality. This review defined several risk factors for mortality in patients with BSIs caused by bacteria, including baseline patient variables (age, malignancy, chronic kidney disease, liver disease, steroid therapy, comorbidity scores, and baseline cognitive and functional status), the setting of infection acquisition (community-acquired, healthcare-associated, or hospital-acquired), factors related to the specific infection (source, pathogen species, and pathogen resistance), the inflammatory response (acute disease severity, respiratory rate, and acute altered mental status), and management parameters (appropriate empirical therapy and source control). However, these findings from general ICUs, which often encompass mixed surgical and medical populations, may not be fully generalizable to the distinct context of a medical ICU. Patients in medical ICUs present a unique challenge; they are typically older, carry a heavier burden of chronic comorbidities, and are more frequently immunocompromised-conditions that inherently elevate their risk for severe infections and poor outcomes. Furthermore, the management of these complex patients often necessitates interventions such as steroid therapy, which itself is a recognized risk factor for infection-related mortality.

Early identification of mortality risk factors is essential for improving patient outcomes. While determinants of mortality in general ICUs have been studied, data specifically focusing on patients with BSIs in a dedicated medical ICUs remain limited. The present study aims to address this gap by examining a homogeneous cohort admitted to a medical ICU under strict, non-surgical criteria. Furthermore, our analysis incorporates the local pathogen ecology, which was dominated by Gram-negative bacteria, to identify context-specific and generalizable risk factors for mortality. Our finding of Gram-negative bacterial predominance and a substantial 1-month mortality (62.3%) aligns with the EUROBACT-2 international cohort study, which identified Gram-negative pathogens as frequent causes of ICU-acquired BSIs and reported a concerning 28-day mortality rate of 37% [9].

The present study aims to identify predictors of mortality among critically ill patients in a medical ICU with ICU-acquired BSIs, where patients are generally older, have more comorbidities, and are more likely to be immunocompromised compared to those in a general ICU [12]. The ability to identify BSI patients at high risk of mortality facilitates early prognostic assessment and informs clinical decision-making, thereby enhancing the potential for life-saving interventions.

## Methods

### Study population

**Fig. 1:**
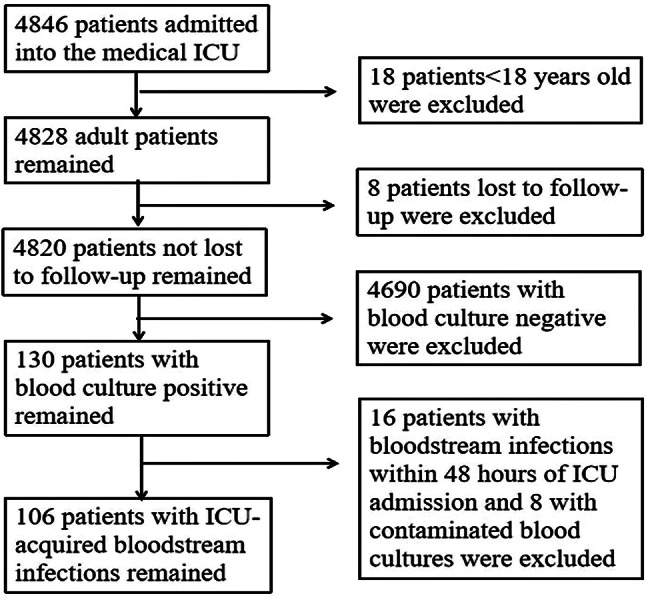
Flow diagram for cohort selection

This single-center, retrospective study included all adult patients with ICU-acquired BSIs in the medical ICU of the Third Xiangya Hospital of Central South University from 1st January 2015 to 31st December 2023. The exclusion criteria were: patients aged < 18 years and patients lost to follow-up. A total of 4846 patients were admitted to the medical ICU during the study period. Of the 4820 patients of age ≥ 18 years and not lost to follow, 106 (2.2%) developed BSIs 48 h after their admission in the medical ICU and were eventually included in the analysis (Fig. 1). The medical ICU only admits critically ill patients from internal medicine departments, and does not admit patients who have undergone surgery in surgical departments to homogenize the patients. Ethical approval for this study was obtained from the Ethics Committee of the Third Xiangya Hospital under approval number 24,307. A waiver of consent was granted by the Ethics Committee due to this retrospective study. This study was conducted in full compliance with the ethical principles outlined in the Declaration of Helsinki for medical research involving human subjects. Participant confidentiality and data protection were maintained throughout the study.

### Definitions

The definition of infection followed the standard criteria established by the European Centre for Disease Prevention and Control [13]. BSIs were defined as the isolation of a bacterium or fungus, excluding specific pathogens (coagulase-negative *Staphylococci*, *Corynebacterium* spp., *Bacillus* spp., *Cutibacterium* spp., non-hemolytic *Streptococci* of the viridans group, *Aerococcus* spp., or *Micrococcus* spp.) from at least one blood culture, accompanied by clinical features consistent with systemic inflammatory response syndrome (https://www.cdc.gov/nhsn/pdfs/pscmanual/4psc_clabscurrent.pdf). ICU-acquired BSIs were defined as occurring 48 h after ICU admission [9]. Septic shock was defined as sepsis with hyperlactacemia and hypotension, necessitating the use of vasopressors [14]. Primary BSI was defined as BSI occurring in patients with no known source of infection (https://www.cdc.gov/nhsn/pdfs/pscmanual/4psc_clabscurrent.pdf). A Gram-negative bacterium was classified as carbapenem-resistant if it was nonsusceptible to at least one carbapenem (imipenem, meropenem, or doripenem) [15]. Antibiotic treatment was considered “appropriate” if the pathogen was identified and its sensitivity profile determined, ensuring the antibiotic used was effective [16]. Deaths were all-cause mortality including in-hospital and post-discharge mortality.

### Microbiologic studies

Samples were collected for clinical bacterial and fungal culture, including blood, sputum, bronchoalveolar lavage fluid, urine, ascites, and catheter tip. Surveillance blood cultures were obtained when patients exhibited abnormal body temperature. A 10-mL blood sample was inoculated into BacT/ALERT brand aerobic and anaerobic culture bottles and then incubated using the BACTEC 9240 automated blood culture system (Becton Dickinson, USA), with microbial and fungal identification via the Bruker mass spectrometry. Bacterial susceptibilities utilized the automated Vitek-2 system (bioMérieux, Marcyl’Etoile, France) and fungal susceptibilities utilized TDR YEAST-AST system (20230816; Hunan Mindray Medical Technology Co., Ltd., China). Antimicrobial susceptibility and the minimum inhibitory concentration were measured by the broth microdilution method. The interpretation of positive blood culture results and the diagnosis of BSI were conducted in accordance with established clinical microbiology standards and guidelines. Briefly, the isolation of a recognized pathogen from one or more blood cultures was considered significant. The growth of common skin contaminants, such as *Staphylococcus epidermidis*, required isolation of the same microorganism from at least two separate blood cultures, supported by clinical evidence of infection, to be deemed a true BSI rather than contamination.

Quality control was performed using *Escherichia coli* ATCC 25,922, *Pseudomonas aeruginosa* ATCC 27,853, *Staphylococcus aureus* ATCC 25,923, and *Candida albicans* ATCC 60,193, which are American Type Culture Collection strains. Results were evaluated according to the NCCLs manual [17]. All antibiotics, except the vancomycin and teicoplanin powders (purchased from Tongtai Co., Guangzhou, China), were products of Oxoid, England. Intermediate susceptibility to antibiotics was regarded as resistance. Only the first episode of BSI was included for patients with multiple BSIs of the same pathogen.

### Data content and acquisition methods

Data were extracted from medical records, including demographics, comorbid conditions, antimicrobial use, laboratory and radiographic results, and clinical management which were potentially related to mortality. All patients were followed for at least 1 month after the onset of BSIs. We conducted telephone follow-ups with all discharged patients. For those who visited the outpatient department after discharge, we accessed their relevant information through the outpatient electronic medical record system. For those who did not come for the visit, we conducted telephone follow-ups to inquire about their survival condition. We obtained the data required for this study from medical records in March and April 2024.

### Statistical analyses

Data are presented as absolute numbers (proportions) for categorical variables and as medians (interquartile range [IQR]) for non-normally distributed variables, or as mean ± standard deviation for normally distributed continuous variables. Associations between mortality and clinical findings were assessed using the χ² test or Fisher’s exact test, as appropriate. The final multivariable model for predictors of mortality was pre-specified based on the conceptual framework encompassing host factors (age, BMI, lymphocyte count, presence of malignancy), disease severity (body temperature, septic shock at onset), and critical therapeutic intervention (appropriate antibiotic treatment), with an odds ratio (OR) and a 95% confidence interval (CI). Both discrimination (the area under the receiver operating characteristic curve with 95%CI) and calibration (slope/intercept) together with the non-significant Hosmer-Lemeshow test of the final model were reported. These analyses were performed using SPSS software, version 26.0 (SPSS, Inc., Chicago, IL), and statistical significance was set at *P* < 0.05, with two-tailed tests. Forest plots were provided to visually summarize adjusted ORs for 1-month mortality risk factors using GraphPad Prism, version 10.6.0 (GraphPad Software, San Diego, CA, USA)

## Results

### General characteristics and prognosis of patients with BSIs

A total of 113 bacterial or fungal isolates were obtained from 106 cases of BSIs in the medical ICU. The frequency of BSIs was 2.2% (106/4820) in the current study. Five patients experienced different types of BSIs twice each (*Klebsiella pneumoniae* and *Klebsiella ozaenae*, *Acinetobacter baumannii* and *P. aeruginosa*, *K*. *ozaenae* and *K. pneumoniae*, *A. baumannii* and *P. aeruginosa*, and *K*. *ozaenae* and *P. aeruginosa*, respectively), and one patient experienced different bacterial infections three times in a row (*A. baumannii*, *Burkholderia cepacia* and *Flavobacterium)*, with the remaining patients undergoing BSIs caused by a single organism.

The study population comprised 71 males (67.0%) and 35 females (33.0%), with a mean age of 64.2 ± 17.1 years. The median body mass index (BMI) was 23.0 (IQR: 21.0–23.0). The two most common comorbidities were lung infections (93.4%) and type 2 diabetes (36.8%), followed by malignancy (21.7%) and chronic obstructive pulmonary disease (19.8%). The lungs were the most frequent source of BSIs (50.0%). Septic shock developed in 71 patients (67.0%) at the onset of BSIs. Among the 106 patients, 81 received corticosteroids. To facilitate comparison, doses were converted to methylprednisolone equivalents based on the following standard equivalence: prednisone 5 mg = methylprednisolone 4 mg = hydrocortisone 20 mg = dexamethasone 0.75 mg. The timing and dosage of methylprednisolone-equivalent therapy were as follows: 55 patients received corticosteroids before the onset of BSIs, with a minimum dose of 24 mg, a maximum of 1760 mg, and a median (IQR) of 160 (53, 360) mg; 44 patients received corticosteroids within 48 h after BSI onset, with a minimum dose of 13.2 mg, a maximum of 240 mg, and a median (IQR) of 53 (27.9, 106) mg; 34 patients received corticosteroids more than 48 h after BSI onset, with a minimum dose of 13.2 mg, a maximum of 1000 mg, and a median (IQR) of 105 (58.3, 185.5) mg. Appropriate antibiotic treatment was prescribed to 51.9% of patients within 48 h of BSI onset. A total of 83 patients required mechanical ventilation, and 26 required renal replacement therapy. The median ICU and hospital lengths of stay were 10.0 (IQR: 4.0-20.3) and 16.0 (IQR: 7.0-28.3) days, respectively. Sixty-six (62.3%) patients died within 1 month after the onset of BSIs. The baseline demographic, clinical, and laboratory characteristics of these 106 patients with BSIs are detailed in Table 1.

**Table 1:** Demographic, laboratory, and clinical variables of 106 patients in medical ICU with BSIs

Characteristics	Value
Demographics	
Age, mean year ± SD	64.2 ± 17.1
Gender, no. of male (%)	71(67.0)
BMI, median (IQR),	23.0(21.0–23.0)
Clinical characteristics, n (%)	
Pulmonary infection	99(93.4)
Type 2 diabetes	39(36.8)
Gastrointestinal bleeding	26(24.5)
Malignancy	23(21.7)
COPD	21(19.8)
COVID-19 infection	11(10.4)
Pneumorrhagia	4(3.8)
Septic shock on the onset of BSIs, n (%)	71(67.0)
Infection-related data	
Type of BSIs, n (%)	
Gram-negative infections	66(62.3)
Gram-positive infections	35(33.0)
Fungal infections	5(4.7)
Source of BSIs, n (%)	
Lungs	53(50.0)
Central line-associated	11(10.4)
Urinary tract	8(7.5)
Skin	4(3.8)
Primary	30(28.3)
Laboratory and clinical parameters	
WBC count on the onset of BSIs, median (IQR), ×10^9^/L	9.9(5.3–15.4)
Lymphocyte count on the onset of BSIs, median (IQR), ×10^9^/L	0.5(0.3–1.1)
Platelet count on the onset of BSIs, median (IQR), ×10^9^/L	129.5(67.5-213.5)
Albumin level on the onset of BSIs, median (IQR), g/L	28.5(24.3–31.8)
Immune globulin level on the onset of BSIs, median (IQR), g/L	26.9(21.9–31.9)
Creatinine on the onset of BSIs, median (IQR), mg/dL	1.0(0.7-2.0)
CRP on the onset of BSIs, median (IQR), mg/L	148.0(54.8-216.3)
PCT on the onset of BSIs, median (IQR), ng/mL	4.8(0.8–14.6)
Body temperature on the onset of BSIs, median (IQR), ℃	38.5(38.0–39.0)
Interventions	
RBC transfusion, median (IQR), units	0(0–3.0)
Immune globulin use after BSIs, n (%)	8(7.5)
Use of thymosin α1, n (%)	4(3.8)
Steroids use, n (%)	81(76.4)
Appropriate antibiotic treatment, n (%)	55(51.9)
Invasive mechanical ventilation, n (%)	83(78.3)
Renal replacement therapy, n (%)	26(24.5)
Outcome	
ICU stay, median (IQR), days	10.0(4.0-20.3)
Hospitalization stay, median (IQR), days	16.0(7.0-28.3)
All-cause mortality within 1 month after BSIs, n (%)	66(62.3)

Abbreviations: BMI, body mass index; BSIs, bloodstream infections; COPD, chronic obstructive pulmonary disease; COVID, Coronavirus disease; CRP, C reactive protein; ICU, intensive care unit; IQR, interquartile range; PCT, procalcitonin; RBC, red blood cell; SD, standard deviation; WBC, white blood cell

Among the 113 organisms isolated, Gram-negative bacteria accounted for 64.6%, followed by Gram-positive bacteria (31.0%). The most frequent pathogens were Enterobacteriaceae (36.3%), including *K. pneumoniae* (25.7%), *E. coli* (6.2%), *K. ozaenae* (3.5%) and *Proteus mirabilis* (0.9%) (Table 2).

**Table 2:** Classification and constituent ratio of pathogens

Bacteria	Strain(*n* = 113)	Constituent ratio(%)
Gram-negative bacteria	73	64.6
* Klebsiella pneumoniae*	29	25.7
* Klebsiella ozaenae*	4	3.5
* Escherichia coli*	7	6.2
* Acinetobacter baumannii*	21	18.6
* Burkholderia cepacia*	4	3.5
* Pseudomonas aeruginosa*	4	3.5
Others	4	3.5
Gram-positive bacteria	35	31.0
* Staphylococcus aureus*	14	12.4
* Staphylococcus hominis*	3	2.7
* Staphylococcus epidermidis*	3	2.7
* Enterococcus faecium*	10	8.8
Others	5	4.4
Fungi	5	4.4
* Candida tropicalis*	2	1.8
* Candida krusei*	1	0.9
* Candida parapsilosis*	1	0.9
* Candida albicans*	1	0.9

### Analysis of the risk factors for 1-month all-cause mortality

Univariate and multivariate analyses were performed to identify potential risk factors for 1-month all-cause mortality following BSIs, with results listed in Table 3. In the univariate analysis, malignancy (*P* = 0.023), carbapenem-resistant Gram-negative BSIs (*P* = 0.008), lymphocyte count < 0.5 × 10⁹/L (*P* = 0.001), immune globulin level < 25 g/L (*P* = 0.007), body temperature ≥ 39℃ (*P* = 0.001), and septic shock (*P* < 0.001) at the onset of BSIs, steroids use (*P* < 0.001), as well as the need for renal replacement therapy (*P* = 0.025) and mechanical ventilation (*P* < 0.001), were associated with mortality. Conversely, the survival group exhibited a higher frequency of BMI ≥ 23 (*P* = 0.012) and appropriate antibiotic treatment after BSI onset (*P* = 0.012) than the death group.

**Table 3:** Univariate analysis of risk factors for 1-month all-cause mortality after BSIs

Variables	Death (66)	Survival (40)	*P*	OR (95% CI)
Male sex, n (%)	45(68.2)	26(65.0)	0.736	
Age, n (%)				
≥ 75 years	25(37.9)	8(20.0)	0.054	
60–74 years	18(27.3)	16(40.0)	0.174	
< 60 years	23(34.8)	16(40.0)	0.594	
BMI, n (%)				
≥ 23	6(9.1)	11(27.5)	0.012	
18.5–22.9	53(80.3)	25(62.5)	0.044	
< 18.5	7(10.6)	4(10.0)	1.000	
Malignancy, n (%)	19(28.8)	4(10.0)	0.023	
COPD, n (%)	14(21.2)	7(17.5)	0.642	
Type 2 diabetes, n (%)	21(31.8)	18(45.0)	0.173	
COVID-19 infection, n (%)	10(15.2)	1(2.5)	0.082	
Pulmonary infection, n (%)	37(56.1)	16(40.0)	0.807	
Gastrointestinal bleeding, n (%)	17(25.8)	9(22.5)	0.706	
Pneumorrhagia, n (%)	5(7.6)	0(0)	0.190	
Gram-negative BSIs, n (%)	44(66.7)	22(55.0)	0.230	
Carbapenem-resistant Gram-negative BSIs, n (%)	32(48.5)	9(22.5)	0.008	
Gram-positive BSIs, n (%)	19(28.8)	16(40.0)	0.234	
Respiratory origin, n (%)	47(71.2)	21(52.5)	0.051	
WBC count at the onset of BSIs < 4 × 10^9^/L, n (%)	14(21.2)	4(10.0)	0.136	
Lymphocyte count at the onset of BSIs, n (%)				
> 1.0 × 10^9^/L	12(18.2)	14(35.0)	0.051	
0.5-1.0 × 10^9^/L	13(19.7)	15(37.5)	0.044	
< 0.5 × 10^9^/L	41(62.1)	11(27.5)	0.001	
Platelet count at the onset of BSIs ≤ 50 × 10^9^/ L, n (%)	16(24.2)	7(17.5)	0.414	
Albumin level at the onset of BSIs < 25 g/L, n (%)	18(27.3)	13(32.5)	0.566	
Immune globulin level at the onset of BSIs < 25 g/L, n (%)	34(51.5)	10(25.0)	0.007	
Creatinine at the onset of BSIs ≥ 2 mg/dL, n (%)	19(28.8)	9(22.5)	0.477	
CRP at the onset of BSIs ≥ 100 mg/L, n (%)	44(66.7)	21(52.5)	0.147	
PCT at the onset of BSIs ≥ 10ng/mL, n (%)	24(36.4)	10(25.0)	0.224	
Body temperature at the onset of BSIs, n (%)				
≥ 39℃	30(45.5)	6(15.0)	0.001	
37.1–38.9℃	29(43.9)	25(62.5)	0.064	
36.0–37.0℃	7(10.6)	9(22.5)	0.097	
Septic shock at onset of BSIs, n (%)	54(81.8)	17(42.5)	< 0.001	
RBC transfusion, n (%)	25(37.9)	13(32.5)	0.773	
Immune globulin use after BSIs, n (%)	6(9.1)	2(5.0)	0.694	
Use of thymosin a1, n(%)	3(4.5)	1(2.5)	0.992	
Use of steroids, n (%)	58(87.9)	23(57.5)	< 0.001	
Appropriate antibiotic treatment after BSIs, n (%)	28(42.4)	27(67.5)	0.012	
Mechanical ventilation, n (%)	59(89.4)	24(60.0)	< 0.001	
Renal replacement therapy, n (%)	21(31.8)	5(12.5)	0.025	

Abbreviations: BMI, body mass index; BSIs, bloodstream infections; CI, confidence intervals; COPD, chronic obstructive pulmonary disease; COVID, Coronavirus disease; CRP, C reactive protein; OR, odds ratios; PCT, procalcitonin; RBC, red blood cell; WBC, white blood cell

The final multivariable model was pre-specified based on the conceptual framework encompassing host factors (age, BMI, lymphocyte count, presence of malignancy), disease severity (body temperature, septic shock at onset), and critical therapeutic intervention (appropriate antibiotic treatment). None of these continuous variables (age, BMI, lymphocyte count, and body temperature) reached conventional statistical significance as independent linear predictors of mortality. We believe this important finding suggests that the association between these factors and outcome is not linear across their entire range but may be driven by a high-risk state beyond a critical threshold. So, based on the World Health Organization’s definition, age was categorized into three groups: <60 years (adults), 60–74 years (younger older adults), and ≥ 75 years (older adults). Only patients aged ≥ 75 years showed a trend toward a significant association with mortality in the chi-square analysis across the three age groups, with a *P* value at the margin of statistical significance (*P* = 0.054). Following the WHO classification for Asian populations, we now model BMI as a categorical variable with four levels: Underweight (< 18.5 kg/m²), normal weight (18.5–22.9 kg/m²), overweight (23.0–24.9 kg/m²), and obesity (≥ 25.0 kg/m²). Given the similar mortality and survival rates between the overweight (23.0–24.9 kg/m²) and obesity (≥ 25.0 kg/m²) categories, and considering the limited number of events in each, these two groups were combined into a single“overweight/obesity (BMI ≥ 23.0 kg/m²)”category for all subsequent statistical analyses to enhance the robustness of comparisons. Based on clinical significance, the lymphocyte count was categorized into three groups: normal (> 1.0 × 10⁹/L), mild reduction (0.5-1.0 × 10⁹/L), and severe reduction (< 0.5 × 10⁹/L). An analysis using multiple categories showed a graded increase in mortality risk only in the most severe category. Since no patients presented with hypothermia, body temperature was categorized into three groups: normothermia (36.0–37.0℃), mild-to-moderate fever (37.1–38.9℃), and high fever (≥ 39℃). Only patients in the high fever group exhibited an increased mortality rate. All these findings reinforce that these risks are threshold-dependent rather than linear. All continuous variables that were ultimately included in the multivariate analysis were categorized and are presented in the Table 3. We eventually did not present continuous analyses in the multivariable analyses, and just investigated the association between age ≥ 75 years, malignancy, BMI ≥ 23, lymphocyte count < 0.5 × 10⁹/L, body temperature ≥ 39℃, septic shock at BSI onset, and appropriate antibiotic treatment after BSI onset and 1-month all-cause mortality. The final model showed good discrimination with an area under the curve of 0.844 (95% CI: 0.769–0.919). The model also demonstrated excellent calibration. The calibration slope was 0.966 (95% CI: 0.927–1.006), indicating no significant deviation from perfect discrimination. The calibration intercept was 0.022 (95% CI: -0.005-0.049), indicating no significant overall over- or under-prediction of risk. Together with the non-significant Hosmer-Lemeshow test (χ² = 3.636, *P* = 0.821), these results confirm that the model is well-calibrated across the entire risk spectrum.

Although carbapenem resistance and pulmonary source are known risk factors, carbapenem resistance was not included to avoid collinearity with antibiotic appropriateness, as its effect is largely mediated through delayed effective therapy. Pulmonary source was not included as a primary variable to maintain model parsimony, given that its prognostic information may be captured by the more direct measure of septic shock. Steroid use was not included in the final multivariate analysis for two primary reasons. First, a significant proportion of patients received only minimal doses, which are unlikely to have a material impact on mortality. Second, and more critically, steroid administration in this cohort was often a clinical response to deteriorating patient condition. Therefore, its use likely served more as a marker of illness severity rather than an independent causative factor for poor outcomes.

**Fig. 2:**
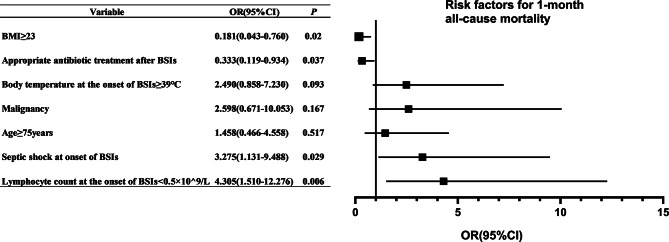
Forest plot illustrating multivariate logistic regression analysis of the independent risk factors for 1-month mortality after the onset of BSIs. Abbreviations: BMI, body mass index; BSIs, bloodstream infections; CI, confidence intervals; OR, odds ratios

The multivariate analysis revealed that lymphocyte count < 0.5 × 10⁹/L [OR = 4.305, 95%CI: 1.510-12.276, *P* = 0.006], and septic shock at BSI onset [OR = 3.275, 95%CI: 1.131–9.488, *P* = 0.029] were associated with 1-month all-cause mortality. Conversely, BMI ≥ 23 [OR = 0.181, 95%CI: 0.043–0.760, *P* = 0.020] and appropriate antibiotic treatment after BSI onset [OR = 0.333, 95%CI: 0.119–0.934, *P* = 0.037] were associated with decreased 1-month all-cause mortality. Age ≥ 75 years [OR = 1.458, 95%CI: 0.466–4.558, *P* = 0.517], malignancy [OR = 2.598, 95%CI: 0.671–10.053, *P* = 0.167], and body temperature ≥ 39℃ [OR = 2.490, 95%CI: 0.858–7.230, *P* = 0.093] were not associated with the increase of 1-month all-cause mortality (Fig. 2).

## Discussions

BSI is a frequent complication among ICU patients worldwide, representing a severe systemic infectious disease caused by bacterial and fungal pathogens. Among the pathogens responsible for BSIs, Gram-negative bacteria are often the most prevalent [9, 18–20]. The predominant pathogens identified in our study included *E. coli*, *Acinetobacter* spp., *Klebsiella* spp., coagulase-negative *staphylococci*, *S. aureus*, and *Streptococcus* spp [9, 21–23]. A previous study evaluating mortality risk factors for BSIs in five adult general ICUs in Italy demonstrated a significant association between *K. pneumoniae* and mortality [24]. Additionally, the link between carbapenem-resistant Gram-negative BSIs and increased mortality has been well established, emphasizing the role of antimicrobial-resistant pathogens in mortality [9]. In our cohort, 2.4% developed BSIs, and mortality reached 62.3% at 1 month, predominantly due to Gram-negative organisms (*K. pneumoniae* and *A. baumannii*). These findings align with previous studies [9, 10, 18, 19, 21–23]. The high mortality observed in our BSI cohort further underscores the severe burden of ICU-acquired infections, which, as reported in a large global JAMA prevalence study, affected over half of all ICU patients and were associated with a sobering 30% hospital mortality [10]. Carbapenem-resistant Gram-negative BSIs were significantly associated with higher mortality in univariate analysis in the present study. Furthermore, we found that carbapenem resistance was strongly associated with a markedly higher rate of inappropriate empirical antibiotic therapy (56.9% vs. 43.1%, *P* < 0.001). Since inappropriate therapy itself was a powerful independent predictor of mortality, its inclusion in the multivariate model likely subsumed the statistical effect of resistance. This suggests that the heightened risk of death from resistant pathogens is principally mediated through the initial administration of ineffective antibiotics, underscoring the critical importance of optimizing empirical therapy in high-risk patients.

Healthcare-associated BSIs, or ICU-acquired BSIs, often stem from primary infections in other anatomical sites, with respiratory and urinary tracts being the most common sources [9, 25]. In particular, pneumonia-related BSIs have been associated with higher 28-day and total mortality rates compared to other types of BSI [9, 25, 26]. The pooled prevalence of catheter-associated BSIs in critically ill patients from contemporary studies ranges from 26.4% to 39.5%, with an overall crude mortality rate of 38.4% [27, 28]. In our study, the respiratory tract was the most common source of secondary BSIs, though no significant association was found between respiratory sources and mortality.

A meta-analysis of 60 studies (15,681 patients) examining biomarkers for mortality prediction in critically ill patients with sepsis found no predictive value for procalcitonin or C-reactive protein, which aligns with our findings [29]. Furthermore, COVID-19 infection and advanced age, have been recognized as major risk factors for increased mortality in ICU patients [17, 30, 31]. However, our study did not identify a significant relationship between these factors and mortality among patients with BSIs. This discrepancy may reflect differences in patient characteristics and the relatively small sample size of our study.

Body temperature is another important factor associated with mortality in BSI patients. A study by Cai et al. revealed that hypothermia was significantly associated with 14-day and 28-day mortality in BSI patients [32]. In contrast, none of patients occurred hypothermia at the onset of BSIs in our present study which found that a body temperature of ≥ 39℃ at the onset of BSIs showed a trend toward a significant association with mortality in the final multivariable model. Higher body temperature reflects a more severe infection and an amplified inflammatory response, which likely contributes to increased mortality [33].

Septic shock is strongly associated with mortality in BSI patients, as shown in our study and supported by many other studies [21, 30, 32, 34]. Septic shock signifies a critical stage in illness severity, and early interventions such as intravenous fluid resuscitation and prompt empirical antibiotic therapy are essential for improving survival [35].

Immunocompromised patients are at heightened risk of poor outcomes in BSI, as their immune response is impaired. In our study, lower lymphocyte counts at the onset of BSIs was associated with 1-month mortality. A recent multicentre, double blinded, placebo controlled phase 3 trial of a cellular immune enhancer, thymosin α1, which could elevate the amount of lymphocytes, showed its potential differential effect on 28 day all-cause mortality in septic patients of ≥ 60 years and patients with diabetes, indicating that a weakened immune system leads to an increase in mortality rate [36]. Reduced lymphocyte counts reflect compromised cellular immunity, exacerbating infection outcomes [37].

BMI has been identified as a predictor of mortality in sepsis, including BSIs. This relationship, however, often follows a counterintuitive pattern known as the “obesity paradox,” where obesity appears to confer a survival advantage. Several studies have shown that a higher BMI is associated with lower mortality compared to a lower BMI in patients with sepsis [38, 39]. Our study adds support to the “obesity paradox” by observing that a higher BMI was associated with lower 1-month mortality in BSI patients [40]. Several theories have been proposed to explain this paradox, including the presence of additional fuel sources in the septic catabolic state, muscle tissue that mitigates harmful effects, adipokines that regulate immune function, and higher leptin levels that protect against severe septic shock [39, 41].

Rapid and appropriate antibiotic therapy is essential for improving the prognosis of patients with BSIs, including those caused by carbapenem-resistant *K. pneumoniae* and other pathogens [1, 22, 30, 42]. However, ineffective empirical antibiotic therapy was found to be a significant contributor to increased mortality, as observed in our study, where only 48.1% of patients received effective empiric therapy [43].

Our study identified several key factors associated with mortality in patients with BSIs from a medical ICU, including host factors (low/normal BMI, severely low lymphocyte count), disease severity (septic shock at onset of BSIs), and critical therapeutic intervention (inappropriate antibiotic treatment). The risk factors identified align with those found in previous studies [11, 21, 30, 32, 34, 37, 38, 39, 43]. However, variations across hospitals, geographic regions, and socioeconomic factors may influence these associations.

The high mortality rate in ICU patients with BSIs underscores the need for measures to reduce BSI incidence and improve patient outcomes. Previous studies have demonstrated that national prevention guidelines and hygiene interventions can significantly reduce BSI rates in ICU settings [44].

In addition, rapid and accurate diagnostic technologies such as whole-genome sequencing or metagenomic next-generation sequencing can facilitate early antimicrobial therapy and optimize patient treatment [1, 26].

The strong association of severe lymphopenia and low/normal BMI with mortality in our cohort highlights their potential utility in risk assessment. First, these parameters, particularly a lymphocyte count < 0.5 × 10⁹/L, could serve to identify patients at heightened risk, who might benefit from more intensive monitoring. Second, our findings provide a rationale for future research to evaluate whether integrating early nutritional support for patients with low/normal BMI or immunomodulatory strategies for those with severe lymphopenia into clinical protocols can improve outcomes. This approach—complementing pathogen-directed therapy with strategies aimed at supporting host defenses—represents a promising but unvalidated avenue for improving care in this vulnerable population.

Despite its limitations, including its single-center, retrospective design, and the relatively small cohorts of patients with ICU-acquired BSIs and non-survivors, this study provides valuable insights into a critically ill yet understudied population. Furthermore, conducted over 9 years in a medical ICU that exclusively admits non-surgical, critically ill internal medicine patients, our research offers important clinical data particularly relevant to an aging population. This patient group is characterized by a higher burden of comorbidities and immunocompromised states compared to those in general wards or mixed ICUs. Furthermore, this study established independent predictors of 1-month mortality in patients with ICU-acquired BSIs by developing a prespecified multivariate model grounded in clinical prior knowledge. The model demonstrated excellent discrimination and calibration, and its findings balance statistical rigor with clinical interpretability, providing empirical evidence for bedside risk stratification.

### Considerations for generalizability

The generalizability of our findings, derived from a single Chinese tertiary hospital, requires contextual interpretation. The pathogen spectrum in our cohort was predominantly Gram-negative, which may reflect epidemiological patterns distinct from Western ICUs, where Gram-positive organisms could be more prevalent. Nevertheless, the pathophysiological insights gleaned from our analysis extend beyond regional specificities. The consistent identification of severe lymphopenia as a marker of immunoparalysis, the fatal impact of septic shock on mortality, the critical role of timely and appropriate antibiotic treatment, and the repeated observation of the “obesity paradox” (with BMI ≥ 23 serving as a factor associated with lower 1-month mortality) all align with well-established principles of sepsis pathophysiology and have been documented across diverse critical care settings. Thus, while local microbiological ecology may vary, our findings provide a rationale for investigating the integration of host immune and nutritional parameters into risk-stratification strategies for BSIs in critically ill adults across different settings.

## Conclusion

This single-center retrospective study identified BMI, lymphocyte count, septic shock, and antibiotic use as factors associated with 1-month mortality in medical ICU patients with ICU-acquired BSIs, providing a basis for risk stratification. The clinical implications of these findings should be framed as directions for future investigation rather than immediate practice changes: First, the strong association with septic shock reinforces the universal imperative for its early recognition and management. Second, the link to antibiotic appropriateness underscores established stewardship goals. Third, the associations of mortality with BMI and lymphopenia suggest the potential value of, and need for prospective studies on, nutritional and immune status assessment in this population. Finally, the high mortality observed reiterates the fundamental importance of robust infection control strategies to prevent BSIs in critically ill patients.

## Data Availability

The datasets used and/or analyzed during the current study are available from the corresponding author upon reasonable request.

## Abbreviations

BSI: Bloodstream infection
ICU: Intensive care unit
IQR: Interquartile range
CI: Confidence interval
BMI: Body mass index
COPD: Chronic obstructive pulmonary disease
COVID: Coronavirus disease
CRP: C reactive protein
PCT: Procalcitonin
RBC: Red blood cell
OR: Odds ratios
SD: Standard deviation
WBC: White blood cell
